# Hippocampal Damage and Atrophy Secondary to Status Epilepticus in a Patient with Schizophrenia

**DOI:** 10.3389/fneur.2017.00024

**Published:** 2017-02-06

**Authors:** Elaine Keiko Fujisao, Nathalia Raquel Cristaldo, Aline Marques da Silva Braga, Paulina Rodrigues Cunha, Seizo Yamashita, Luiz Eduardo Betting

**Affiliations:** ^1^Departamento de Neurologia, Psicologia e Psiquiatria, Faculdade de Medicina de Botucatu, Universidade Estadual Paulista (UNESP), Botucatu, Brazil; ^2^Departamento de Doenças Tropicais e Diagnósticos por Imagem, Faculdade de Medicina de Botucatu, Universidade Estadual Paulista (UNESP), Botucatu, Brazil

**Keywords:** temporal lobe epilepsy, hippocampal sclerosis, neuroimaging, schizophrenia, EEG

## Abstract

A 59-year-old man was admitted with respiratory tract infection, compromised conscience and generalized tonic–clonic seizures. His medical history included schizophrenia diagnosis, for which he had been being treated since he was 27 years old. EEG disclosed non-convulsive status epilepticus. A magnetic resonance image (MRI) acquired 3 days later showed increased left hippocampal volume with hyperintensity on T2-weighted and FLAIR sequences. After being treated with antibiotics and antiepileptic medications, the patient’s condition improved. A follow-up MRI showed reduction of the left hippocampus. The relationship between epilepsy and schizophrenia is not yet clear. This case illustrates this interaction. Hippocampal atrophy may have been caused by environmental aggression in the present patient with schizophrenia, perhaps in association with a predisposing genotype.

## Background

Hippocampal atrophy (HA) is often seen with mesial temporal lobe epilepsy (MTLE), the most prevalent form of focal epilepsy in adults (1). Typically, patients with MTLE suffer from refractory seizures and may have a history of prolonged febrile seizures dating back to childhood (1). In adults, HA may be secondary to an acute insult. We report magnetic resonance image (MRI) findings disclosing hippocampal swelling associated with status epilepticus followed by HA 3 months later in an adult previously diagnosed with schizophrenia.

## Introduction

A 59-year-old man was admitted to the emergency room with compromised consciousness for 1 day. Relatives reported he had been experiencing coughing, prostration, and fever during the week before admission and generalized tonic–clonic seizures 3 days earlier, after which he became unconscious. His medical history included schizophrenia diagnosis, for which he had been treated since he was 27 years old. He was taking risperidone 1 mg/day and biperiden 2 mg/day with satisfactory control of symptoms. Physical examination revealed that the patient had a tonic posture of the limbs and deviation of the head and eyes. Non-convulsive status epilepticus (NCSE) was suspected.

**Figure 1:**
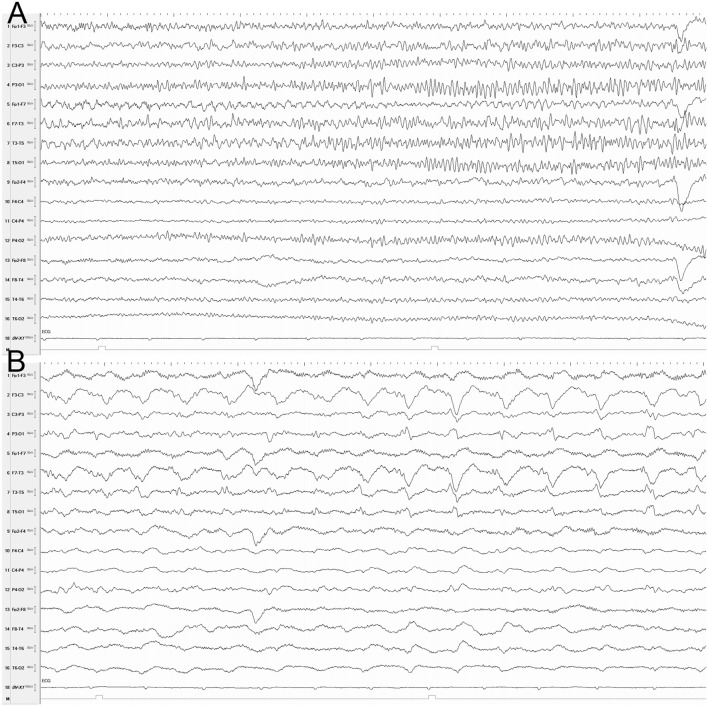
**EEG (longitudinal montage) showing a seizure at onset (A) and 30 s later (B)**. **(A)** Rhythmic theta activity, mainly in the left temporal region. **(B)** Rhythmic delta and sharp waves in the left hemisphere. Sensitivity, 10 µV; time constant: 0.3 s; high filter, 30 Hz; Notch filter, 60 Hz; 10 s/page.

EEG was performed with a 32-channel recorder (Nihon Kohden, Tokyo, Japan). Electrodes were positioned according to the 10–20 international system of electrode placement and with additional Silverman’s anterior temporal electrodes. EEG disclosed recurrent seizures, and the NCSE was terminated with midazolan and phenytoin (Figure 1).

Blood samples showed elevated C-reactive protein (4.0 mg/L), leukocytosis (14,000 white blood cells/μL; 77% neutrophils, 12% lymphocytes, and 11% monocytes), and very elevated creatine phosphokinase (12,000 U/L); all other parameters were normal. Cerebrospinal fluid and chest X-rays were also normal. A computerized tomography scan of the brain on the day of admission showed no alterations.

**Figure 2:**
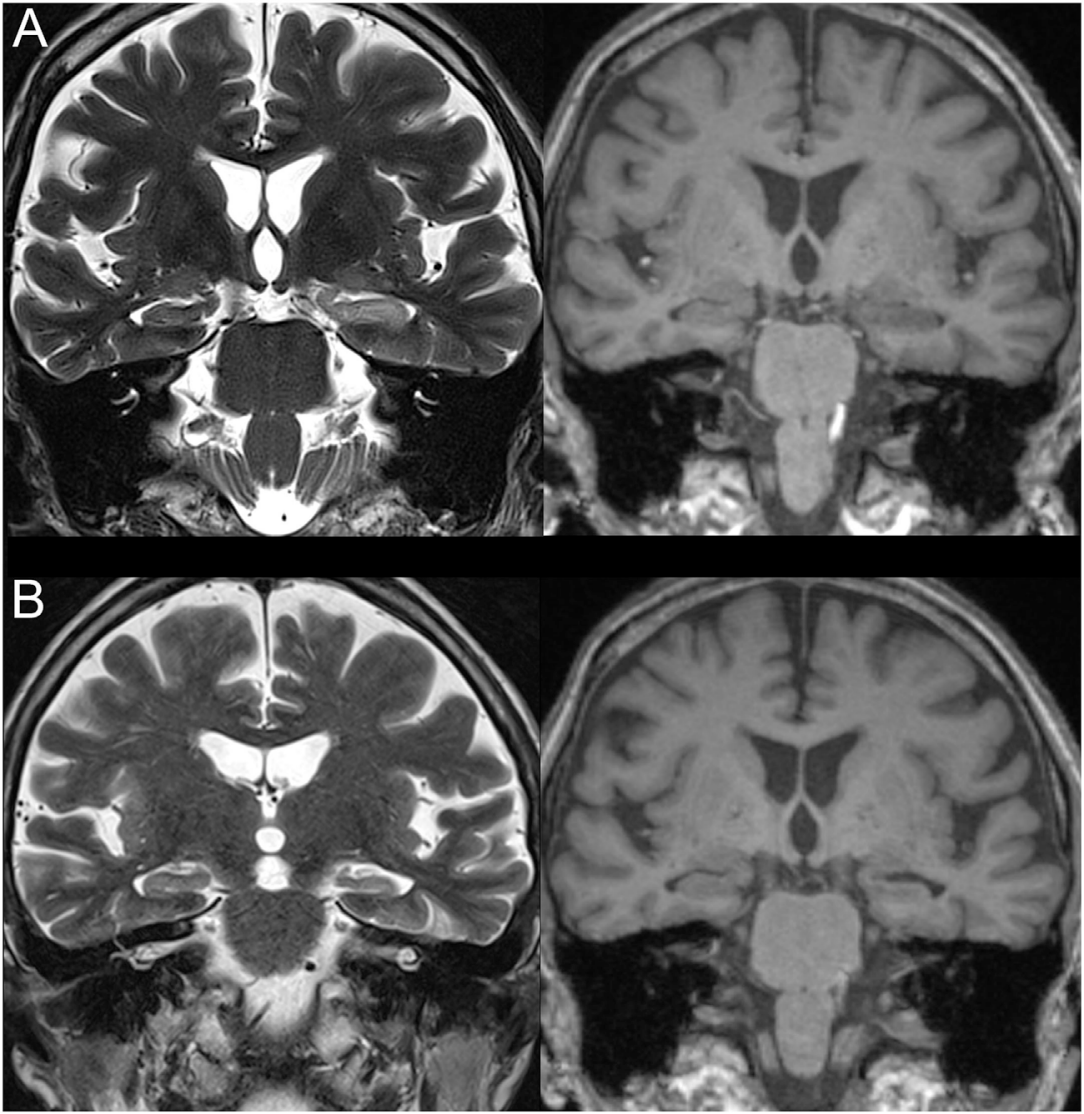
**T1- (right) and T2- (left) weighted magnetic resonance imaging images (coronal slices)**. **(A)** Increased left hippocampal volume with increased T2 and decreased T1 signals. **(B)** Reduced left hippocampal volume without signal changes.

An MRI was acquired using a 3 T scanner (Siemens, Verio, Erlangen, Germany) with a 12-channel head array coil 3 days later. Coronal slices with 3-mm thickness perpendicular to the long axis of the hippocampus were included to evaluate temporal lobe abnormalities. MRI showed increased left hippocampal volume with hyperintensity on T2-weighted and FLAIR sequences (Figure 2A). After being treated with ceftriaxone, the patient’s responsiveness and overall clinical condition improved. Corticosteroids and antiviral therapy were not used. A follow-up MRI 3 months after discharge showed reduction of the left hippocampus with enlargement of the left ventricle temporal horn (Figure 2B). Six months after discharge, the patient remains seizure-free using valproate 1,500 mg/day.

## Discussion

Psychotic symptoms occur in 2–7% of patients with epilepsy (2). There is controversy regarding whether schizophrenia is a risk factor for epilepsy (3). Prevalence rates of psychosis in epilepsy may differ according to seizure-symptom onset interval. When the interval is short (post-ictal psychosis), estimates indicate that 2–11% of patients with epilepsy have comorbid psychosis. Interictal psychosis may be present in 3–9% of epilepsy cases (2).

The relationship between epilepsy and schizophrenia is not yet clear. Gelisse et al. (4) showed that the prevalence of epilepsy and acute symptomatic seizures is relatively low in patients with schizophrenia, compared to the general population. In a study comparing epilepsy patients receiving disability benefits with people with other somatic diseases, Stefansson et al. (5) found that 6.2% of the epilepsy group had a psychosis diagnosis, particularly schizophrenia and paranoid states, compared to only 2.3% of the control group, suggesting that the association between seizures and schizophrenia may involve common genetic or environmental causes (3). In a large 9-year Danish study (*N* = 558,958), an increased risk of schizophrenia (44%) was found in people with a history of febrile seizures. Both febrile seizures and epilepsy were associated with a more than doubled risk of schizophrenia (6). Likewise, MTLE has also been related strongly with febrile seizures (7).

Magnetic resonance image findings in patients with schizophrenia have included enlarged ventricles and whole-brain volume reduction, especially in the prefrontal cortex, superior temporal gyrus, hippocampus, and amygdala (3). Similar to MTLE, amygdalo–hippocampal circuitry might play an important role in the development of schizophrenia (8). Meanwhile, several neuroimaging studies have demonstrated acute swelling of the hippocampus after prolonged febrile seizures. These findings are related predominantly to prolonged febrile seizures in childhood (9, 10).

Genetic predisposition is an important causal factor of febrile seizures and HA (11). Several genes have been associated with susceptibility to schizophrenia (3). Abnormal neurodevelopmental hypothesis may explain the common occurrence of epilepsy and schizophrenia (3).

Subacute progressive decrease in the level of consciousness is the typical presentation of encephalitis. Infectious and autoimmune encephalitis are important differential diagnosis, which were considered in the current case. Most of infectious encephalitis are viral, and psychiatric symptoms are common early in the course of autoimmune encephalitis (12). Unfortunately, viral panel in the CSF and antibody testing were not performed. The hypotheses of viral and autoimmune encephalitis were weakened by the long duration of the psychiatric symptoms and the substantial response to antibiotics without the significant use of corticosteroids or antiviral therapy. Probably toxic-metabolic encephalopathy caused by pulmonary bacterial infection was the trigger to the status epilepticus.

In subjects with MTLE, the development of HA remains under investigation (13). The cause–consequence relationship between seizures and HA still have to be clarified. In adults, prolonged seizure and status epilepticus may lead to pathologically documented HA (14). In the present case, history of subtle or febrile seizures was negative, but they cannot be completely excluded. Therefore, in this setting, the interaction between genetical background with schizophrenia phenotype, age, and the acute infectious disease probably were decisive for the HA pathogenesis.

## Concluding Remarks

Epilepsy and schizophrenia may share susceptibility. Epilepsy is associated with an increased risk of schizophrenia-like symptoms. This case illustrates this possible relationship. HA may have been caused by environmental aggression in the present patient with schizophrenia, perhaps in association with a predisposing genotype.

## Ethics Statement

Retrospective case report (exempted). Informed consent was signed for publication. Patient was not identified.

## Author Contributions

NC, AB, and PC made substantial contributions to the collection, analysis and interpretation of the data, and revising the manuscript. SY made substantial contributions to the analysis and interpretation of the data and revising the manuscript. EF and LB made substantial contributions to the conception and design of the study, collection, analysis and interpretation of data, as well as to the preparation of the first draft and further revisions of the manuscript. All authors approved the final version of the manuscript.

## Conflict of Interest Statement

EF, NC, AB, PC, SY, and LB report no disclosures.

## References

[1] Engel J Jr. Mesial temporal lobe epilepsy: what have we learned? Neuroscientist (2001) 7(4):340–52. 10.1177/107385840100700410 11488399

[2] Araújo-Filho GM Rosa VP Yacubian ELT Psychiatric disorders in epilepsy: a proposal for classification by the ILAE commission on neuropsychiatry. J Epilepsy Clin Neurophysiol (2008) 14(3):119–23.

[3] Cascella NG Schretlen DJ Sawa A. Schizophrenia and epilepsy: is there a shared susceptibility? Neurosci Res (2009) 63(4):227–35. 10.1016/j.neures.2009.01.002 19367784 PMC2768382

[4] Gelisse P Somalian JC Genton P. Is schizophrenia a risk factor for epilepsy or acute symptomatic seizures? Epilepsia (1999) 40:1566–71. 10.1111/j.1528-1157.1999.tb02041.x 10565584

[5] Stefansson SB Olafsson E Hauser WA. Psychiatric morbidity in epilepsy: a case controlled study of adults receiving disability benefits. J Neurol Neurosurg Psychiatry (1998) 64:238–41. 10.1136/jnnp.64.2.238 9489538 PMC2169938

[6] Vestergaard M Pedersen CB Christensen J Madsen KM Olsen J Mortensen PB. Febrile seizures and risk of schizophrenia. Schizophr Res (2005) 73:343–9. 10.1016/j.schres.2004.07.004 15653280

[7] Malmgren K Thom M Hippocampal sclerosis – origins and imaging. Epilepsia (2012) 53(Suppl 4):19–33. 10.1111/j.1528-1167.2012.03610.x 22946718

[8] Harrison PJ. The neuropathology of schizophrenia. A critical review of the data and their interpretation. Brain (1999) 122(Pt 4):593–624. 10.1093/brain/122.4.593 10219775

[9] VanLandingham KE Heinz ER Cavazos JE Lewis DV. Magnetic resonance imaging evidence of hippocampal injury after prolonged focal febrile convulsions. Ann Neurol (1998) 43:413–26. 10.1002/ana.410430403 9546321

[10] Scott RC Gadian DG King MD Chong WK Cox TC Neville BG Magnetic resonance imaging findings within 5 days of status epilepticus in childhood. Brain (2002) 125:1951–9. 10.1093/brain/awf202 12183341

[11] Cendes F Febrile seizures and mesial temporal sclerosis. Curr Opin Neurol (2004) 7(2):161–4. 10.1097/00019052-200404000-00013 15021243

[12] Lancaster E. The diagnosis and treatment of autoimmune encephalitis. J Clin Neurol (2016) 12(1):1–13. 10.3988/jcn.2016.12.1.1 26754777 PMC4712273

[13] Kobayashi E Li LM Lopes-Cendes I Cendes F. Magnetic resonance imaging evidence of hippocampal sclerosis in asymptomatic, first-degree relatives of patients with familial mesial temporal lobe epilepsy. Arch Neurol (2002) 59(12):1891–4. 10.1001/archneur.59.12.1891 12470176

[14] Kumar G Mittal S Moudgil SS Kupsky WJ Shah AK. Histopathological evidence that hippocampal atrophy following status epilepticus is a result of neuronal necrosis. J Neurol Sci (2013) 334(1–2):186–91. 10.1016/j.jns.2013.08.016 23992920

